# Association between baseline hemoglobin levels and pathological complete response in 404 female breast cancer patients undergoing neoadjuvant therapy in Western Guangdong region: a retrospective cohort study

**DOI:** 10.3389/fonc.2026.1699201

**Published:** 2026-03-06

**Authors:** Lixia Li, Jiacheng Yang, Huijie Deng, Biyao Zhang, Guangyu Zheng, Yuzhi Wu, Ziheng Yang, Yuzhou Wang, Zhongcheng Liang, Yuanqi Zhang

**Affiliations:** 1 Cancer Hospital, Affiliated Hospital of Guangdong Medical University, Zhanjiang, China; 2 Medical Oncology Department II, Central Hospital of Guangdong Nongken, Zhanjiang, China; 3 Department of Oncology, Maoming People’s Hospital, Maoming, China; 4 Department of Breast Surgery, Affiliated Hospital of Guangdong Medical University, Zhanjiang, China

**Keywords:** breast cancer, hemoglobin, neoadjuvant therapy, pathological complete response, prognosis

## Abstract

**Background:**

Breast cancer is the most common female malignancy globally and a leading cause of cancer-related death. In China, an estimated 350,000 new cases were reported in 2022, with more than 65% of patients aged ≤35 years diagnosed at stage II or higher. Neoadjuvant therapy (NAT) is recommended by NCCN guidelines for operable breast cancer with clinical stage ≥T2, node-positive, HER2-positive, or triple-negative disease. Achieving pathological complete response (pCR) after NAT is associated with improved event-free survival (EFS), but benefits vary widely, underscoring the need for predictive biomarkers.

**Objective:**

To evaluate the association between baseline hemoglobin (Hb) levels and pCR and EFS in female breast cancer patients from Western Guangdong, China.

**Method:**

This retrospective study included 404 invasive breast cancer patients who received NAT between 2014 and 2024. Multivariate logistic regression and Cox models were used to assess Hb’s relationship with pCR and EFS. Dose-response was analyzed using restricted cubic splines. Patients were stratified by Hb tertiles: Q1 (<123 g/L), Q2 (123–133 g/L), Q3 (>133 g/L). Kaplan-Meier curves were generated for survival analysis.

**Results:**

The pCR rate was 37.1%. Each 1 g/L increase in Hb was associated with a 2% increase in pCR probability (OR = 1.02, 95% CI: 1.01–1.04). Subgroup analyses confirmed consistency across molecular subtypes, menopausal status, and BMI. Multivariate Cox analysis showed each 1 g/L Hb increase reduced event risk by 2% (HR = 0.98, 95% CI: 0.97–0.99). During median follow-up, 83 (20.5%) patients had events; EFS differed significantly across Hb tertiles (log-rank P < 0.001).

**Conclusion:**

Baseline Hb demonstrates a significant linear positive correlation with pCR and EFS, supporting its potential as a simple, low-cost biomarker for predicting NAT benefit in breast cancer patients.

## 1 Introduction

F Globally, breast cancer not only has the highest number of new cases among female cancers but also ranks as one of the leading causes of cancer-related deaths (1). The latest data from China indicate that the estimated number of new cases in 2022 exceeded 350,000. More than 65% of breast cancer patients aged ≤35 years were diagnosed at clinical stage II or higher, and this proportion is even higher among middle-aged and elderly patients (2, 3). Current NCCN guidelines recommend that neoadjuvant therapy (NAT) should be considered the preferred strategy for all operable breast cancer patients with clinical stage ≥T2, lymph node-positive, HER2-positive, or triple-negative breast cancer (TNBC) (4). Previous data have shown that patients who achieve pathological complete response (pCR) after neoadjuvant therapy have significantly better event-free survival (EFS) than those who do not (5). Therefore, neoadjuvant therapy has become an important treatment strategy for Chinese female breast cancer patients requiring downstaging surgery. However, even within the NCCN-recommended population for neoadjuvant therapy, treatment benefits remain heterogeneous (4). Thus, identifying biomarkers that can predict the benefit of NAT is crucial.

The hypoxic tumor microenvironment (TME), primarily mediated by hypoxia-inducible factor (HIF), has been demonstrated to enhance tumor aggressiveness and reduce sensitivity to various treatments (6–8). Hemoglobin (Hb), the primary carrier of oxygen, has levels that directly influence the oxygen delivery capacity to tissues. Consequently, baseline Hb level, as a key indicator of the body’s oxygen-carrying capacity, may affect the degree of tumor hypoxia through the HIF pathway, thereby participating in the regulation of tumor biological behavior and sensitivity to NAT (9). Previous studies have also shown that low Hb levels or anemia are associated with poorer survival outcomes (such as EFS, PFS, OS) in various solid tumors, including breast cancer (10–12). Recent research further suggests that baseline Hb levels may serve as a positive predictive biomarker for response to immune checkpoint inhibitor therapy (13). Furthermore, pre-treatment anemia in breast cancer has been reported to be associated with a poorer response to NAT and lower pCR rates (14, 15). Therefore, baseline Hb level could be a potential biomarker for predicting pathological response (pCR) to neoadjuvant therapy.

However, previous studies investigating the association between Hb and response to NAT have primarily focused on European and Northern Chinese populations. Moreover, these studies often simplified Hb into a dichotomous variable (anemic *vs*. non-anemic) (14, 15). Research quantifying the association between baseline Hb levels as a continuous variable and the rate of pathological complete response (pCR) after neoadjuvant therapy in a Southern Chinese female breast cancer population remains lacking. Therefore, this study aims to utilize retrospective medical record data to investigate the association between pre-NAT baseline Hb levels and post-treatment pCR rates in female breast cancer patients from Western Guangdong, China. It further seeks to explore the association between Hb levels and event-free survival (EFS).

## 2 Methods

### 2.1 Patients and treatments

The primary focus of this study is the relationship between hemoglobin levels and the pCR rate in female breast cancer patients receiving neoadjuvant therapy. According to literature (5), the pCR rate for breast cancer patients undergoing neoadjuvant therapy is approximately 32.5%. Based on preliminary research, the number of variables expected to be included in the model is 8. The sample size was calculated using the 10 events per variable (EPV) formula: $n=\frac{10\times EPV}{0.325}$, resulting in n=247 cases. Accounting for a 10% rate of loss to follow-up and refusal, a minimum of 272 subjects were ultimately required.

This retrospective study included 587 female patients with histologically confirmed invasive breast cancer who were treated between 2014 and 2024 at the Affiliated Hospital of Guangdong Medical University and Guangdong Provincial Nongken Central Hospital. Patients from the Affiliated Hospital of Guangdong Medical University were identified from a specific database within the hospital’s big data intelligence platform. This platform is a research system that organizes, links, and structures data in real-time from multiple clinical business systems, enabling multidimensional big data queries. Data from Guangdong Provincial Nongken Central Hospital were obtained from its traditional electronic medical record system.

**Figure 1:**
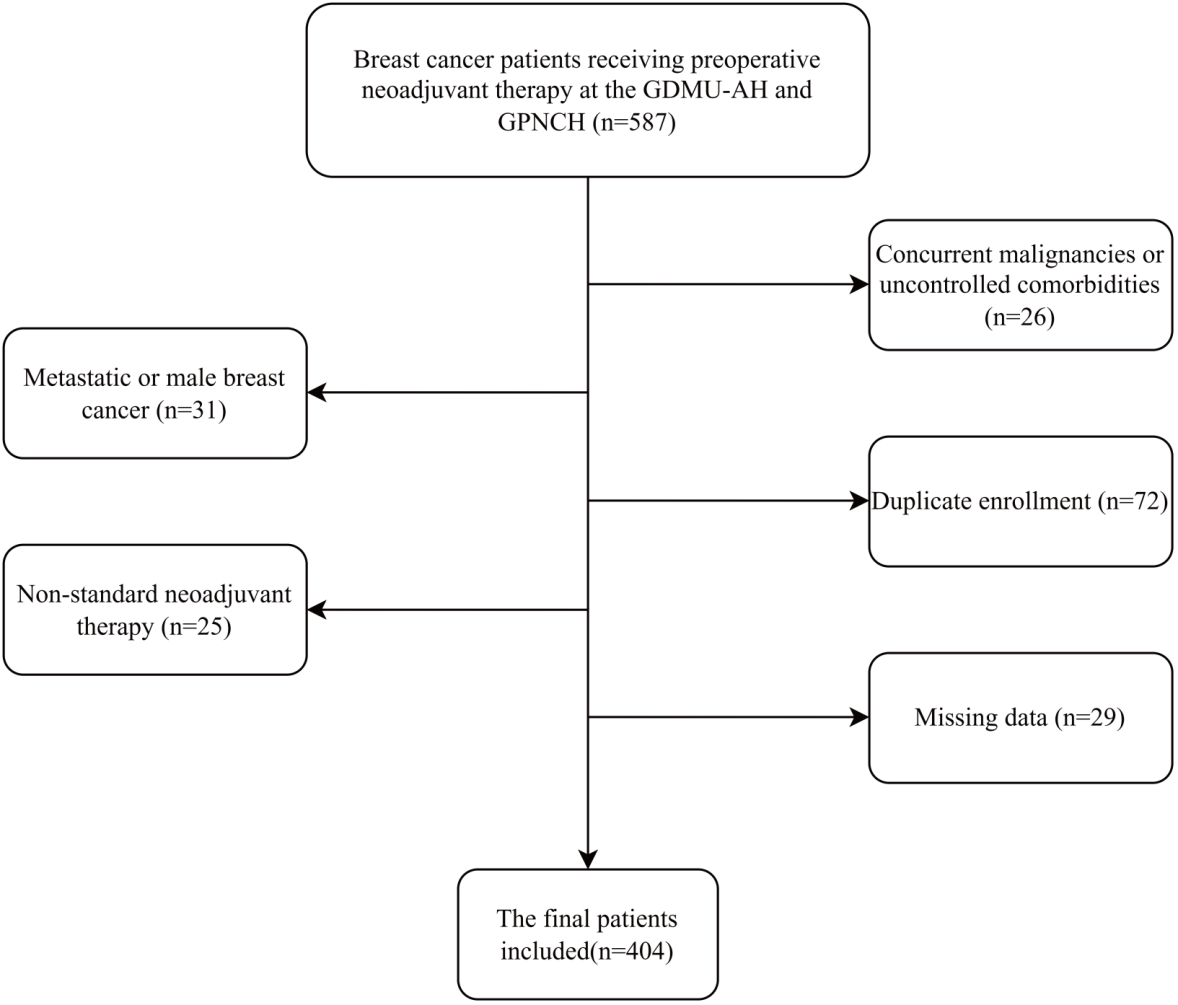
Flow chart of the patient selection. GDMU-AH, Guangdong Medical University Affiliated Hospital; GPNCH, Guangdong Provincial Nongken Central Hospital.

All patients completed NAT and surgery (if feasible) between April 2014 and December 2024. The exclusion criteria for all subjects were as follows: (1) patients with distant metastases or male breast cancer; (2) patients with uncontrolled liver disease, autoimmune diseases, or hematological disorders; (3) patients with significant acute bleeding; (4) pregnant patients or those with concomitant cancers; (5) patients who did not complete at least 2 cycles of standard NAT; and (6) patients with missing data. Ultimately, 404 patients met the criteria for analysis (**Figure 1**).

Pretreatment clinical data were retrospectively collected, including age, menopausal status, tumor size, lymph node involvement, clinical stage (staged according to the 8th edition of the American Joint Committee on Cancer TNM staging system) (16), estrogen receptor (ER) status, progesterone receptor (PR) status, human epidermal growth factor receptor 2 (HER2) status, molecular subtype (classified based on hormone receptor and HER2 status), Ki-67 index, and treatment regimen details. All patients received at least 2 cycles of NAT. All HER2-positive patients received at least one targeted agent. Patients with HR-/HER2- and HR+/HER2- disease received standard anthracycline and taxane-based chemotherapy regimens.

### 2.2 Clinical outcome

Patients were followed until loss to follow-up, the study cutoff date (December 31, 2024), or death. The primary outcome was pathological complete response (pCR), and the secondary outcome was event-free survival (EFS). Follow-up was conducted through inpatient medical records, outpatient visit records, and telephone interviews. EFS was defined as the time interval from the initiation of NAT to the first occurrence of any of the following events: disease progression precluding surgical treatment, local or distant recurrence, loss to follow-up, the study cutoff date (December 31, 2024), or death, whichever occurred first.

### 2.3 Bioinformatics and statistical analyses

Demographic and laboratory data were obtained from the big data platform or traditional electronic medical records. Baseline demographic and anthropometric data included age, sex, height, weight, history of prior diseases, family history of cancer, estrogen receptor (ER) status, progesterone receptor (PR) status, human epidermal growth factor receptor 2 (HER-2) status, breast cancer stage, treatment regimen, and tumor size. Body mass index (BMI) was calculated as weight divided by height squared (kg/m²).BMI classification refers to the WHO standards: Underweight (<18.5 kg/m²), Normal (18.5–24.9 kg/m²), Overweight (≥25.0 kg/m²) (17). Hemoglobin (Hb) levels were obtained from the first measurement taken within the first 7 days after hospital admission and were expressed in g/L, consistent with conventional practice. To ensure that baseline Hb levels were not influenced by recent medical interventions or underlying conditions, we excluded patients who had received blood transfusions, iron therapy, or erythropoietin within the 2 weeks prior to admission. Furthermore, we reviewed medical records for conditions that could potentially lower Hb levels (e.g., rheumatoid arthritis, chronic kidney disease, severe active infections) and excluded such patients from the analysis if these conditions were present.

The determination of estrogen receptor (ER) and progesterone receptor (PR) status followed the guidelines of the American Society of Clinical Oncology (ASCO) and the College of American Pathologists (CAP). Immunohistochemical (IHC) staining of ≥1% of cell nuclei was considered positive, while staining of <1% was considered negative. HER-2 status was classified as positive if IHC showed 3+ staining, and negative if IHC staining was 0 or 1 +. For cases with IHC 2+ staining, further evaluation of HER-2 gene status by *in situ* hybridization (ISH) was required, where ISH positivity defined HER-2 positivity and ISH negativity defined HER-2 negativity. The Ki67 labeling index was assessed as the percentage of tumor cells with nuclear staining and was analyzed as a continuous variable.

Breast cancer molecular subtypes were categorized based on hormone receptor (HR) and HER2 expression as follows: HR+/HER2-; HR+/HER2+; HR-/HER2+; and HR-/HER2- (18–20). pCR was defined as the absence of any residual invasive tumor lesions in both breast tissue and lymph nodes (ypT0/is ypN0) (21). Tumor staging was performed according to the 8th edition of the American Joint Committee on Cancer (AJCC) guidelines (16).

All patients were divided into three groups (Q1, Q2, Q3) based on tertiles of Hb levels. Continuous variables are presented as mean ± standard deviation or median and interquartile range (IQR), with comparisons between groups performed using the Kruskal-Wallis test. Categorical variables are described using frequencies (percentages), with comparisons between groups made using the chi-square test or Fisher’s exact test. In the comparison of baseline characteristics between the included patients and the patients excluded due to missing data, standardized mean differences were calculated to quantify the differences between the groups.

Logistic regression models were used to analyze the association between Hb levels and pCR rate. Cox proportional hazards regression models were employed to analyze the association between Hb levels and event-free survival (EFS). Effect sizes are expressed as odds ratios (OR), hazard ratios (HR), and corresponding 95% confidence intervals (95% CI). Trend tests were conducted using the Wald test for continuous variables. Restricted cubic splines (RCS) were used to explore the relationship between Hb levels (as a continuous variable) and pCR rate.

Survival data were described using the Kaplan-Meier method, and survival curves were compared using the log-rank test. A *P*-value <0.05 was considered statistically significant. All statistical analyses were performed using R statistical software version 4.4.2.

## 3 Results

### 3.1 Patient characteristics

This study included a total of 404 breast cancer patients from Western Guangdong, China, who received preoperative neoadjuvant therapy at two hospitals (**Figure 1**). The baseline characteristics of the patients are shown in **Table 1**. The median age of the cohort was 49 years (interquartile range, 42–55 years), with the majority being premenopausal (235, 58.17%). Most patients had normal weight (225, 55.69%) or were overweight (152, 37.62%). The predominant clinical tumor stage was IIIA (149, 36.88%), and the most common molecular subtype was HR+/HER2- (164, 40.59%). Pathological complete response (pCR) was achieved in 150 patients (37.1%). The comparison of baseline characteristics between patients with and without missing data showed good balance (SMD < 0.1) for most variables, a few tumor-related characteristics (clinical stage, HER2 status, molecular subtype, T stage) demonstrated greater differences (**Supplementary Table 1**). However, given the very small sample size of the missing data group, we believe the impact of these differences on the primary conclusions is limited.

**Table 1:** The baseline characteristics of the study population.

Variables	Total (n = 404)	The tertiles of the hemoglobin			*P*
		Q1 (n = 129)	Q2 (n = 136)	Q3 (n = 139)	
Age, M (Q_1_, Q_3_)	49.00 (42.00, 55.00)	48.00 (43.00,53.00)	49.00 (41.75,55.25)	50.00 (42.00,57.00)	0.455
Height, M (Q_1_, Q_3_)	158.00 (155.00, 160.00)	157.00 (155.00,160.00)	158.00 (155.00,160.00)	157.00 (155.00,160.00)	0.072
Weight, M (Q_1_, Q_3_)	57.00 (52.00, 62.62)	57.00 (51.00,61.50)	57.25 (52.00,63.15)	57.00 (52.00,64.50)	0.336
Ki67, M (Q_1_, Q_3_)	40.00 (30.00, 60.00)	40.00 (30.00,60.00)	40.00 (30.00,60.00)	40.00 (30.00,60.00)	0.655
Hemoglobin, M (Q_1_, Q_3_)	129.00 (119.00, 137.00)	114.00 (106.00,118.00)	129.00 (126.00,131.00)	140.00 (137.00,144.00)	**<.001**
Menopausal Status, n(%)					0.060
N	235 (58.17)	86 (66.67)	74 (54.41)	75 (53.96)	
Y	169 (41.83)	43 (33.33)	62 (45.59)	64 (46.04)	
BMI, n(%)					**0.028**
Normal	225 (55.69)	75 (58.14)	72 (52.94)	78 (56.12)	
Over	152 (37.62)	46 (35.66)	48 (35.29)	58 (41.73)	
Under	27 (6.68)	8 (6.20)	16 (11.76)	3 (2.16)	
Clinical Stage, n(%)					0.205
I_IIA	33 (8.17)	5 (3.88)	14 (10.29)	14 (10.07)	
IIB	94 (23.27)	29 (22.48)	27 (19.85)	38 (27.34)	
IIIA	149 (36.88)	46 (35.66)	55 (40.44)	48 (34.53)	
IIIB	41 (10.15)	19 (14.73)	11 (8.09)	11 (7.91)	
IIIC	87 (21.53)	30 (23.26)	29 (21.32)	28 (20.14)	
Subtype, n(%)					0.159
HR-/HER2-	61 (15.10)	16 (12.40)	23 (16.91)	22 (15.83)	
HR-HER2+	98 (24.26)	40 (31.01)	24 (17.65)	34 (24.46)	
HR+HER2-	164 (40.59)	52 (40.31)	62 (45.59)	50 (35.97)	
HR+HER2	81 (20.05)	21 (16.28)	27 (19.85)	33 (23.74)	
T, n(%)					0.284
1	21 (5.20)	3 (2.33)	9 (6.62)	9 (6.47)	
2	193 (47.77)	60 (46.51)	65 (47.79)	68 (48.92)	
3	134 (33.17)	41 (31.78)	47 (34.56)	46 (33.09)	
4	56 (13.86)	25 (19.38)	15 (11.03)	16 (11.51)	
N, n(%)					0.728
0	46 (11.39)	10 (7.75)	17 (12.50)	19 (13.67)	
1	175 (43.32)	56 (43.41)	56 (41.18)	63 (45.32)	
2	96 (23.76)	33 (25.58)	34 (25.00)	29 (20.86)	
3	87 (21.53)	30 (23.26)	29 (21.32)	28 (20.14)	
ER, n(%)					0.374
0	165 (40.84)	56 (43.41)	49 (36.03)	60 (43.17)	
1	239 (59.16)	73 (56.59)	87 (63.97)	79 (56.83)	
PR, n(%)					**0.038**
0	203 (50.25)	74 (57.36)	57 (41.91)	72 (51.80)	
1	201 (49.75)	55 (42.64)	79 (58.09)	67 (48.20)	
HER2, n(%)					0.606
0	86 (21.29)	27 (20.93)	31 (22.79)	28 (20.14)	
1	52 (12.87)	13 (10.08)	20 (14.71)	19 (13.67)	
2	103 (25.50)	32 (24.81)	39 (28.68)	32 (23.02)	
3	163 (40.35)	57 (44.19)	46 (33.82)	60 (43.17)	
pCR, n(%)					**0.022**
Y	150 (37.13)	36 (27.91)	53 (38.97)	61 (43.88)	
N	254 (62.87)	93 (72.09)	83 (61.03)	78 (56.12)	
Hypertension History, n(%)					0.440
N	371 (91.83)	118 (91.47)	128 (94.12)	125 (89.93)	
Y	33 (8.17)	11 (8.53)	8 (5.88)	14 (10.07)	
Diabetes History, n(%)					0.828
N	389 (96.29)	124 (96.12)	132 (97.06)	133 (95.68)	
Y	15 (3.71)	5 (3.88)	4 (2.94)	6 (4.32)	
CHD History, n(%)					0.900
N	397 (98.27)	127 (98.45)	133 (97.79)	137 (98.56)	
Y	7 (1.73)	2 (1.55)	3 (2.21)	2 (1.44)	
Hepatitis History, n(%)					0.792
N	382 (94.55)	123 (95.35)	129 (94.85)	130 (93.53)	
Y	22 (5.45)	6 (4.65)	7 (5.15)	9 (6.47)	
Family Cancer History, n(%)					0.539
N	399 (98.76)	128 (99.22)	133 (97.79)	138 (99.28)	
Y	5 (1.24)	1 (0.78)	3 (2.21)	1 (0.72)	

BMI, body mass index; CHD, coronary heart disease; ER, estrogen receptor; HER2, human epidermal growth factor receptor 2; HR, hormone receptor; IQR, inter-quartile range; pCR, pathological complete response; PR, progesterone receptor; T, clinical tumour (TNM) stage; N, clinical nodal (TNM) stage; HDL, high-density lipoprotein; Fib, fibrinogen; Cr, creatinine; Cysc, cystatin c. Notes: Pathological complete response (pCR): Defined as the absence of any residual invasive tumor in both the breast and lymph nodes (ypT0/is ypN0) (21).Molecular subtypes: Classified based on hormone receptor and HER2 expression status (18–20). Estrogen Receptor (ER) and Progesterone Receptor (PR) status: Positivity was defined as immunohistochemical (IHC) nuclear staining in ≥1% of tumor cells (18). HER2 status: Positivity was defined as an IHC score of 3 +. A score of 0 or 1+ was considered negative. Cases with an IHC score of 2+ required further *in situ* hybridization (ISH) testing, with ISH positivity defining HER2 positivity (19). Clinical Stage: Performed according to the 8th edition of the American Joint Committee on Cancer (AJCC) staging manual (16). Body Mass Index (BMI): Categories defined as Underweight (<18.5 kg/m²), Normal weight (18.5–24.9 kg/m²), and Overweight (≥25.0 kg/m²) according to the World Health Organization (WHO) criteria (17). Hemoglobin (Hb) tertiles: Q1 (<123 g/L), Q2 (123–133 g/L), Q3 (>133 g/L). The cut-off values were derived from the 33.3rd and 66.7th percentiles of the baseline Hb distribution in this study cohort. Ki67 index: The Ki67 labeling index was assessed as the percentage of tumor cells with nuclear staining and was analyzed as a continuous variable. Bold values indicate statistical significance (P < 0.05).

Furthermore, in **Table 1**, we performed a subgroup analysis by dividing patients into three groups based on tertiles of baseline hemoglobin levels: Q1 (<123 g/L, n = 129), Q2 (123–133 g/L, n = 136), and Q3 (>133 g/L, n = 139). No statistically significant differences were observed among the three groups in terms of age, height, weight, Ki-67 index, or most tumor characteristics (T/N stage, ER/HER2 status, clinical stage, molecular subtype) (*P* > 0.05). However, statistically significant differences were found in BMI, PR status, and pCR status (*P* < 0.05). Detailed data in the table revealed a stepwise increase in pCR rate with higher hemoglobin levels: 27.9% in Q1, 39.0% in Q2, and 43.9% in Q3 (*P* = 0.022).

### 3.2 Dose–response relationship between baseline hemoglobin and pathological complete response

Univariate logistic regression (**Table 2**) analysis demonstrated a positive association between hemoglobin levels and pCR. For the continuous variable analysis: OR = 1.02 (95% CI 1.01 to 1.04; *P* = 0.005). For the tertile comparisons: Q2 *vs* Q1: OR = 1.65 (95% CI 0.98 to 2.76; *P* = 0.058); Q3 *vs* Q1: OR = 2.02 (95% CI 1.21 to 3.36; *P* = 0.0077).

After adjusting for potential confounders (menopausal status, age, BMI, T/N stage, ER status, PR status, HER2 status, and Ki-67 index), the positive association between hemoglobin and pCR remained significant (**Table 2**). For the continuous variable: adjusted OR = 1.02 (95% CI 1.01 to 1.04; *P* = 0.010). For the tertile comparisons: Q2 *vs* Q1: adjusted OR = 2.73 (95% CI 1.42 to 5.26; *P* = 0.003); Q3 *vs* Q1: adjusted OR = 2.56 (95% CI 1.37 to 4.79; *P* = 0.003). Trend tests were significant in both the crude model (*P* for trend = 0.007; OR = 1.03, 95% CI 1.01 to 1.05) and the adjusted model (*P* for trend = 0.003; OR = 1.04, 95% CI 1.01 to 1.06).

**Table 2:** Logistics analyses of Hemoglobin and pathological complete response.

Factors	Crude model		Adjusted model	
	*P*	OR (95%CI)	*P*	OR (95%CI)
Hemoglobin	**0.005**	1.02 (1.01 ~ 1.04)	**0.010**	1.02 (1.01 ~ 1.04)
Hemoglobin Q3				
Q1(<123g/L)		1.00 (Reference)		1.00 (Reference)
Q2(123~133 g/L)	0.058	1.65 (0.98 ~ 2.76)	**0.003**	2.73 (1.42~ 5.26)
Q3(>133 g/L)	**0.007**	2.02 (1.21 ~ 3.36)	**0.003**	2.56 (1.37 ~ 4.79)
P for trend	**0.007**	1.03 (1.01 ~ 1.05)	**0.003**	1.04 (1.01 ~ 1.06)

CI, confidence interval; OR, odds ratio.

Adjusted model: Adjusted for menopausal status, age, BMI, T stage, N stage, ER status, PR status, HER2 status, Ki67.

BMI, Under(<18.5 kg/m²), Normal (18.5–24.9 kg/m²), Over(≥25.0 kg/m²). Bold values indicate statistical significance (P < 0.05).

**Figure 2:**
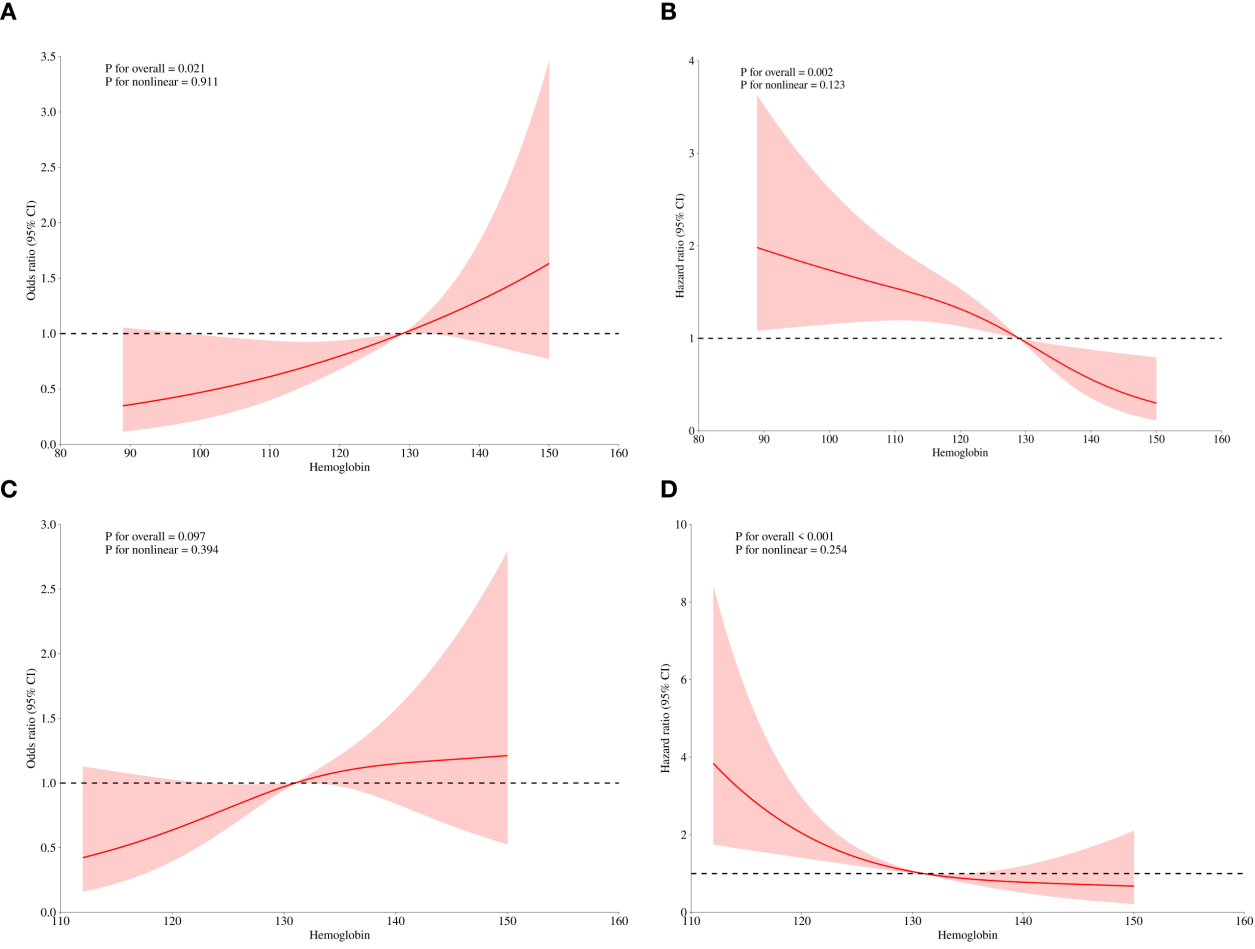
Association between hemoglobin level and pathological complete response rate. Covariates: menopausal status, age, BMI, T stage, N stage, ER status, PR status, HER2 status, Ki67.A. the restricted cubic spline (RCS) graphs of all patients. **(B)** the Cox RCS graph of all patients. **(C)** the restricted cubic spline (RCS) graphs of non-anemic patients. **(D)** the Cox RCS graph of non-anemic patients.

The relationship between hemoglobin (as a continuous variable) and pCR was further explored using restricted cubic splines (RCS). **Figure 2** shows that after adjusting for confounders (menopausal status, age, BMI, T/N stage, ER, PR, HER2 status, and Ki-67), a significant linear relationship was observed between hemoglobin levels and the probability of achieving pCR (*P* for nonlinearity = 0.911). This significant linear association persisted even after excluding patients with hemoglobin levels <110 g/L (*P* for nonlinearity = 0.394).

Furthermore, as shown in **Table 3**, after excluding patients with hemoglobin <110 g/L, logistic regression analyses continued to show a significant positive association in both the crude model (*P* = 0.018; OR = 1.03, 95% CI 1.01 to 1.05) and the adjusted model (*P* = 0.043; OR = 1.03, 95% CI 1.01 to 1.05).

**Table 3:** Analysis after exclusion of patients with hemoglobin levels <110 g/L.

(A)					
Factors	Crude model			Adjusted model	
	*P*	OR (95%CI)		*P*	OR (95%CI)
Hb(≥110g/L)	**0.018**	1.03 (1.01 ~ 1.05)		**0.043**	1.03 (1.01 ~ 1.05)

(B)					
Factors	Crude model			Adjusted model	
	*P*	HR (95%CI)		*P*	HR (95%CI)
Hb(≥110g/L)	**<.001**	0.95 (0.92 ~ 0.97)		**<.001**	0.95 (0.92 ~ 0.98)

CI, confidence interval; HR, hazard ratio; OR, odds ratio; Hb, hemoglobin.

Adjusted model: Adjusted for menopausal status, age, BMI, T stage, N stage, ER status, PR status, HER2 status, Ki67.

BMI, Under(<18.5 kg/m²), Normal (18.5–24.9 kg/m²), Over(≥25.0 kg/m²).After excluding 46 patients with baseline hemoglobin <110 g/L, logistic regression was performed for pathological complete response (pCR) and Cox proportional-hazards analysis for event-free survival (EFS) in the remaining 358 patients. Bold values indicate statistical significance (P < 0.05).

### 3.3 Subgroup analysis

**Figure 3:**
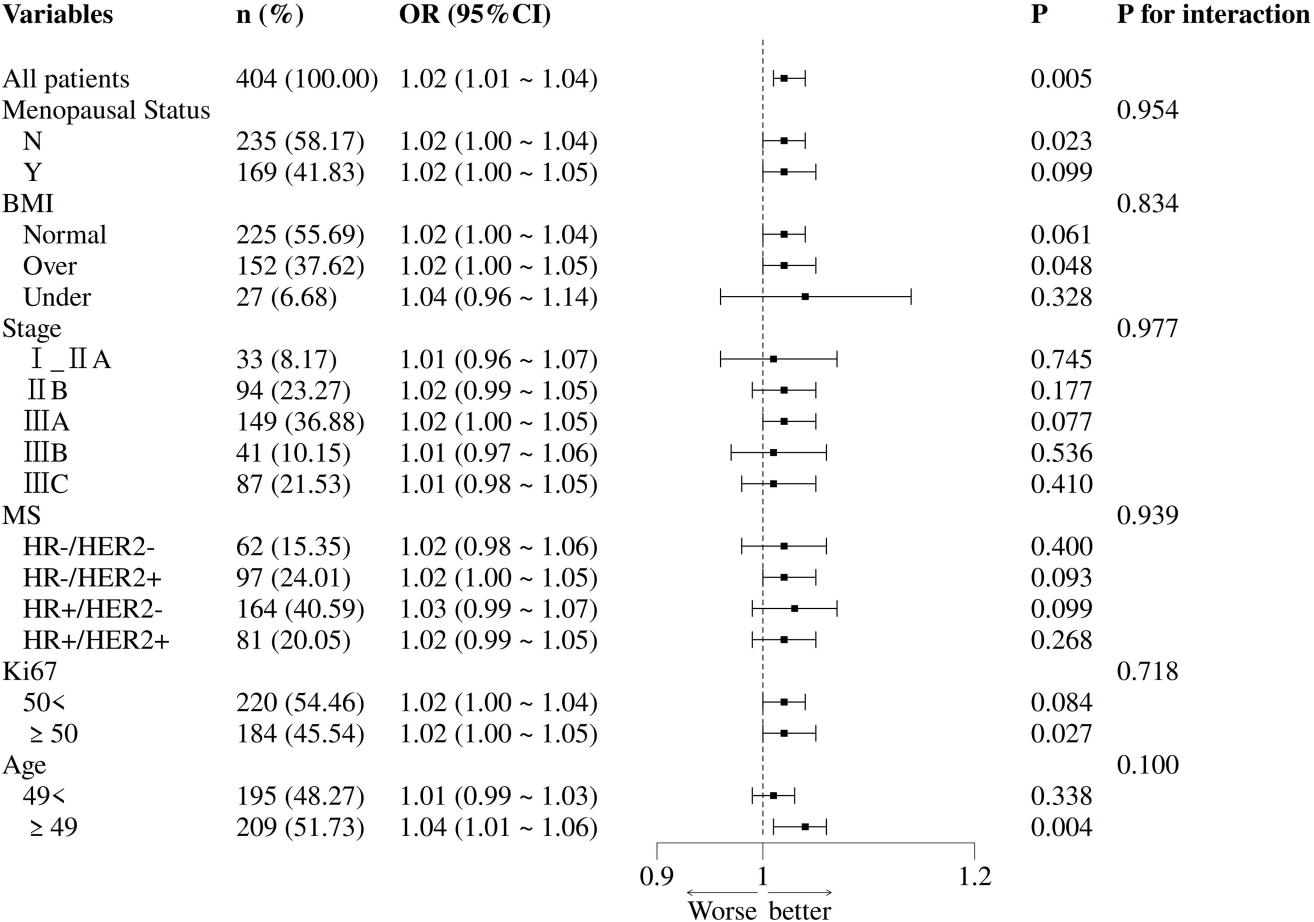
Subgroup analysis of the association between hemoglobin level and pathological complete response rate. Hemoglobin was treated as a continuous variable.

In the subgroup analysis of hemoglobin and pCR (**Figure 3**), this positive association trend remained consistent across all subgroups, and no statistically significant interaction effects were observed in any subgroup. The trends were consistent between premenopausal (OR = 1.02 [1.00–1.04]) and postmenopausal (OR = 1.02 [1.00–1.05]) women. Similar consistent trends were observed across BMI categories: normal weight (OR = 1.02 [1.00–1.04]), overweight (OR = 1.02 [1.00–1.05]), and underweight (OR = 1.04 [0.96–1.14]).

Across clinical stages, all point estimates of OR were greater than 1: Stage I_IIA (1.01 [0.96–1.07]); Stage IIB (1.02 [0.99–1.05]); Stage IIIA (1.02 [1.00–1.05]); Stage IIIB (1.01 [0.97–1.06]); Stage IIIC (1.01 [0.98–1.05]). Similarly, across molecular subtypes, all point estimates were above 1: HR-/HER2- (1.02 [0.98–1.06]); HR-/HER2+ (1.02 [1.00–1.05]); HR+/HER2- (1.03 [0.99–1.07]); HR+/HER2+ (1.02 [0.99–1.05]).

In the Ki-67 subgroups, results were similar for both Ki-67 < 50% (OR = 1.02 [1.00–1.04]) and Ki-67 ≥ 50% (OR = 1.02 [1.00–1.05]). In the age subgroup, patients aged ≥49 years appeared to derive slightly greater benefit (OR = 1.04 [1.01–1.06]), but the interaction test was not statistically significant (*P* = 0.100).

### 3.4 Hemoglobin and event-free survival

**Figure 4:**
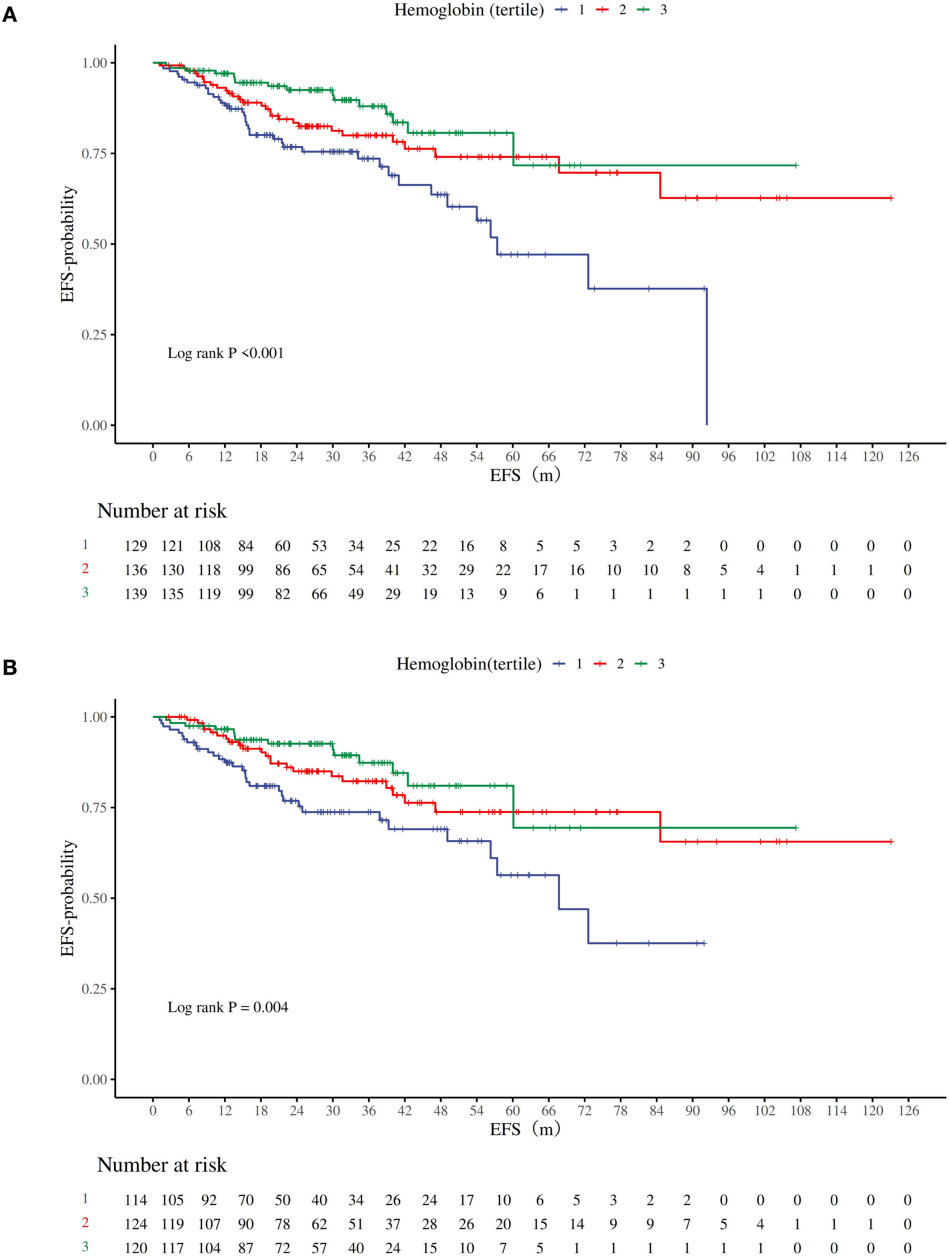
Kaplan–Meier curves for breast cancer patients stratified by hemoglobin tertile. Hemoglobin tertile: Q1(<123 g/L), Q2(123~133 g/L), Q3(>133 g/L). **(A)** the Kaplan–Meier curves of all patients. **(B)** the Kaplan–Meier curves of non-anemic patients (Hb ≥ 110g/L).

A total of 83 events (20.54%) were recorded in the overall study population. When patients were stratified by baseline hemoglobin tertiles, Kaplan-Meier curves showed significant differences in EFS among the three groups (log-rank P < 0.001, **Figure 4**). The Q1 group (<123 g/L) had the lowest 5-year EFS rate, with a rapid decline in the number at risk. The Q3 group (>137 g/L) exhibited the highest curve position, indicating the greatest EFS benefit.

Prior to the Cox analysis, the proportional hazards (PH) assumption test indicated that most variables satisfied the PH assumption. The PH test for hemoglobin yielded a p-value of 0.16, showing no evidence of violation. The PH test for age yielded a p-value of 0.04, suggesting a moderate time-varying effect (**Supplementary Table 2**). However, subsequent sensitivity analyses based on age (**Supplementary Table 4**, **Supplementary Table 5B**) demonstrated that the risk estimates for hemoglobin remained consistently stable, indicating that this minor deviation did not affect the primary conclusions.

Univariate Cox model analysis (**Table 4**) revealed that higher hemoglobin levels were associated with a lower risk of disease progression: Q2 *vs* Q1: HR = 0.54 (95% CI 0.33–0.89; *P* = 0.015); Q3 *vs* Q1: HR = 0.36 (95% CI 0.20–0.65; *P* <.001). After adjusting for menopausal status, age, BMI, T/N stage, ER, PR, HER2 status, and Ki-67 index, the multivariate Cox regression model remained significant: Q2 *vs* Q1: adjusted HR = 0.47 (95% CI 0.28–0.80; *P* = 0.006); Q3 *vs* Q1: adjusted HR = 0.34 (95% CI 0.19–0.63; *P* <.001). Trend tests were significant in both the crude model (*P* for trend <.001; HR = 0.96, 95% CI 0.94–0.98) and the adjusted model (*P* for trend <.001; adjusted HR = 0.96, 95% CI 0.94–0.98).

**Table 4:** Cox analyses of hemoglobin and EFS.

Factors	Crude model		Adjusted model	
	*P*	HR (95%CI)	*P*	HR (95%CI)
Hemoglobin	**<.001**	0.98 (0.96 ~ 0.99)	**<.001**	0.98 (0.97 ~ 0.99)
Hemoglobin Q3				
Q1(<123 g/L)		1.00 (Reference)		1.00 (Reference)
Q2(123~133 g/L)	**0.015**	0.54 (0.33 ~ 0.89)	**0.006**	0.47 (0.28 ~ 0.80)
Q3(>133g/L)	**<.001**	0.36 (0.20 ~ 0.65)	**<.001**	0.34 (0.19 ~ 0.63)
P for trend	**<.001**	0.96 (0.94 ~ 0.98)	**<.001**	0.96 (0.94 ~ 0.98)

EFS, event-free survival, CI, confidence interval; HR, hazard ratio.

Adjusted model: Adjusted for menopausal status, age, BMI, T stage, N stage, ER status, PR status, HER2 status, Ki67.

BMI, Under(<18.5 kg/m²), Normal (18.5–24.9 kg/m²), Over(≥25.0 kg/m²). Bold values indicate statistical significance (P < 0.05).

The relationship between hemoglobin (as a continuous variable) and EFS was further explored using Cox restricted cubic splines (Cox-RCS). The analysis showed that after adjusting for confounders (menopausal status, age, BMI, T/N stage, ER, PR, HER2 status, and Ki-67), there was a significant linear relationship between increasing baseline hemoglobin levels and improved EFS (*P* for nonlinearity = 0.123). This significant linear association persisted even after excluding patients with hemoglobin levels <110 g/L (*P* for nonlinearity = 0.254).

Furthermore, as shown in **Table 2**, after excluding patients with hemoglobin <110 g/L, Cox models continued to show a significant association in both the crude model (*P* <.001; HR = 0.95, 95% CI 0.92–0.97) and the adjusted model (*P* <.001; adjusted HR = 0.95, 95% CI 0.92–0.98). Similarly, after excluding early censored cases (59 cases censored within the first 12 months), the trend of results remained consistent, as detailed in **Supplementary Table 5** and **Supplementary Figure 2**.

## 4 Discussion

This study is the first to investigate the relationship between pre-neoadjuvant therapy baseline hemoglobin levels and pathological complete response (pCR) in a female breast cancer population from Western Guangdong, China. Breaking through the traditional dichotomous analysis method, we employed restricted cubic splines (RCS) to reveal a linear relationship between them. Furthermore, the robustness of this relationship was confirmed by excluding clinically anemic patients (Hb <110 g/L). Additionally, through survival analysis, we discovered that higher baseline hemoglobin levels were also significantly associated with longer event-free survival (EFS), demonstrating its role as an independent prognostic factor validated in this cohort of female breast cancer patients from the Western Guangdong region.

Our findings are consistent with previous reports (14, 15). Compared to prior studies, our research breaks through the limitations of the traditional dichotomous classification (anemic/non-anemic). The results demonstrate that a continuous increase in hemoglobin levels is associated with a higher pCR rate, and this relationship exhibits a linear trend in the restricted cubic splines analysis (P for nonlinearity = 0.911). Moreover, after excluding patients with hemoglobin levels below 110 g/L, the positive association between increasing baseline hemoglobin levels and higher pCR rates remained significant in the logistic regression analysis (*P* = 0.043; OR = 1.03, 95% CI 1.01–1.05).

In the subgroup analyses, the positive association between hemoglobin levels and pCR was consistent across all pre-specified subgroups (menopausal status, BMI, clinical stage, molecular subtype, Ki-67), and no significant interactions were observed (*P* > 0.05). Notably, in the molecular subtype analysis, key subtypes such as HR-/HER2+ (OR = 1.02) and HR+/HER2- (OR = 1.03) also showed positive trends. This suggests that the value of Hb as a biomarker may not be constrained by the intrinsic molecular characteristics of breast cancer.

In the secondary outcome analysis, this study also confirmed that higher hemoglobin levels were associated with a lower risk of disease progression, demonstrating risk stratification capability in Kaplan-Meier curves. These findings align with previous studies reporting the association between anemia and poor prognosis in cancer patients (10–13). Compared to prior research, our study further adjusted for covariates and excluded patients with values below the clinical reference threshold (Hb <110 g/L), yet the significant association between hemoglobin and EFS persisted (*P* <.001; HR = 0.95, 95% CI 0.92–0.98).

Recent studies have indicated that in pan-cancer populations receiving immunotherapy, subgroups with higher baseline hemoglobin levels showed significantly longer overall survival (OS) and progression-free survival (PFS) compared to those with lower levels. This association between hemoglobin levels and immune checkpoint inhibitor (ICI) treatment outcomes appears to be independent of established biomarkers for immunotherapy response (such as TMB, PD-L1, and MSI expression) (13). Previous research has also suggested that low hemoglobin levels predict poor prognosis in radiotherapy cohorts for cancers such as anal cancer and nasopharyngeal carcinoma (22, 23).

Our study cohort, predominantly consisting of female breast cancer patients whose adjuvant therapies primarily included radiotherapy, chemotherapy, and targeted therapy, complements these previous findings and further supports the clinical prognostic value of hemoglobin levels across different cancer types and treatment modalities.

Our study found that as baseline hemoglobin (Hb) levels increase, the rate of pathological complete response (pCR) shows a linear improvement, and baseline Hb can independently predict event-free survival (EFS). However, the underlying biological mechanism remains unclear. We hypothesize that the core of this phenomenon may lie in the subtle relationship between Hb levels and the hypoxic tumor microenvironment (TME): low circulating Hb levels reflect reduced blood oxygen-carrying capacity, which may exacerbate intratumoral hypoxia and promote sustained activation of hypoxia-inducible factor-1α (HIF-1α). Elevated HIF-1α levels can enhance tumor cell growth, metastasis, invasiveness, and drug resistance by modulating genes encoding glycolytic enzymes, angiogenic signaling molecules, and apoptosis/stress response factors (6–8, 24).

Our findings identify hemoglobin as a potential biomarker for optimizing neoadjuvant therapy strategies, offering the advantages of wide accessibility and low cost. However, further prospective studies are needed to validate these results. Additionally, several limitations of this study warrant cautious interpretation: 1. The retrospective design, though adjusted for multiple variables, may still be influenced by residual confounding factors. 2. The study population was recruited over a long time span and exclusively from two hospitals in Western Guangdong, which may limit the generalizability of the conclusions. 3. Due to data constraints, we were unable to analyze the impact of changes in Hb levels during treatment on pCR. 4. There is a lack of direct evidence linking baseline hemoglobin levels to the hypoxic microenvironment. 5. The Hb levels in our study population rarely exceeded 150 g/L, so the applicability of our findings to populations with very high Hb levels remains uncertain.

## 5 Conclusions

This study demonstrates that hemoglobin levels are significantly positively correlated with pCR rates and longer EFS, and this association exhibits a linear relationship. Hemoglobin levels may serve as a potential biomarker for predicting benefit from neoadjuvant therapy (NAT).

## Data Availability

The raw data supporting the conclusions of this article will be made available by the authors, without undue reservation.
