# Supplementary figures and images for: Association between baseline hemoglobin levels and pathological complete response in 404 female breast cancer patients undergoing neoadjuvant therapy in Western Guangdong region: a retrospective cohort study
Source: Front Oncol. 2026 Mar 6;16:1699201. doi: 10.3389/fonc.2026.1699201 (PMC13002421; doi:10.3389/fonc.2026.1699201)

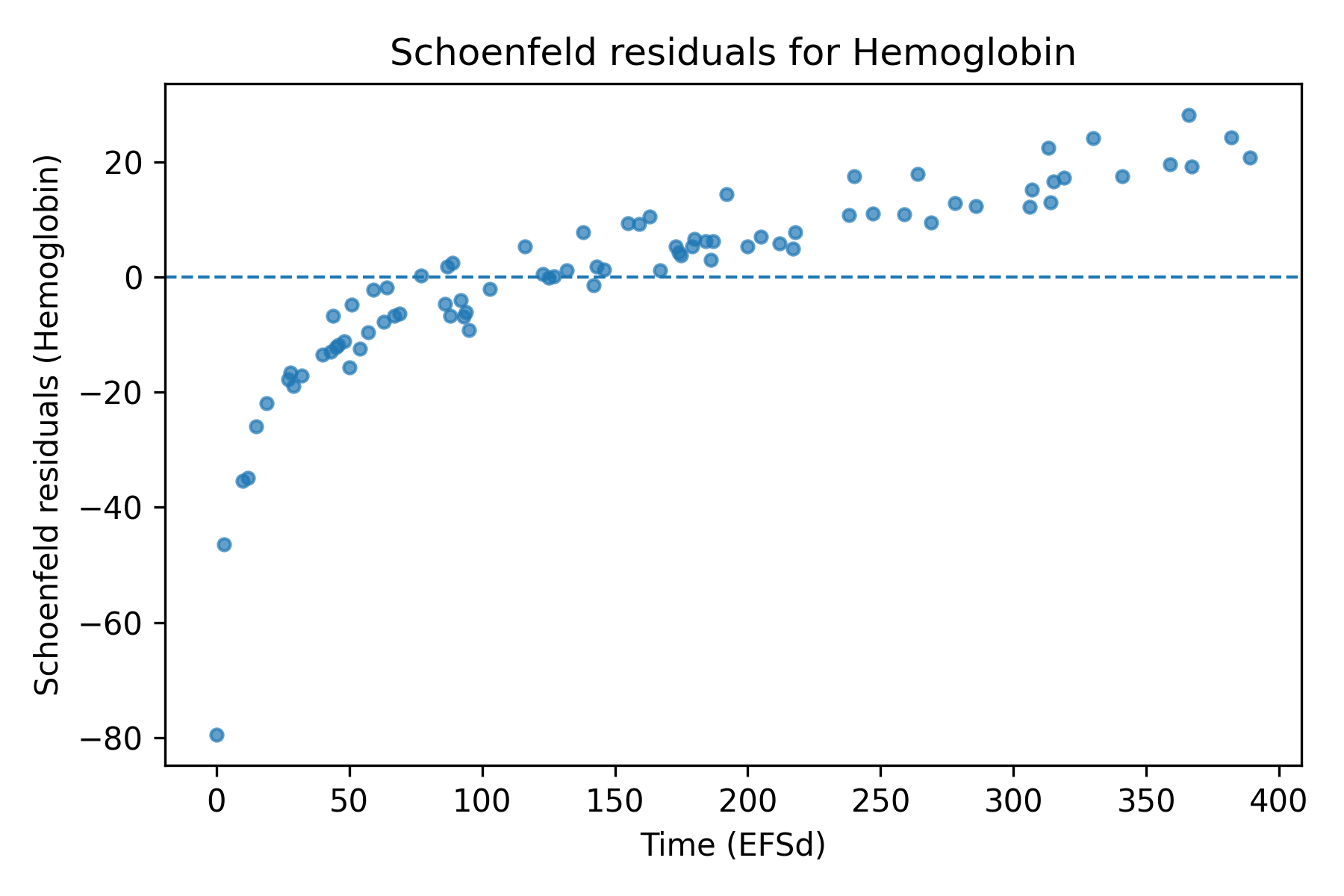

Supplement: Supplementary file 1 [file Image1.tif]

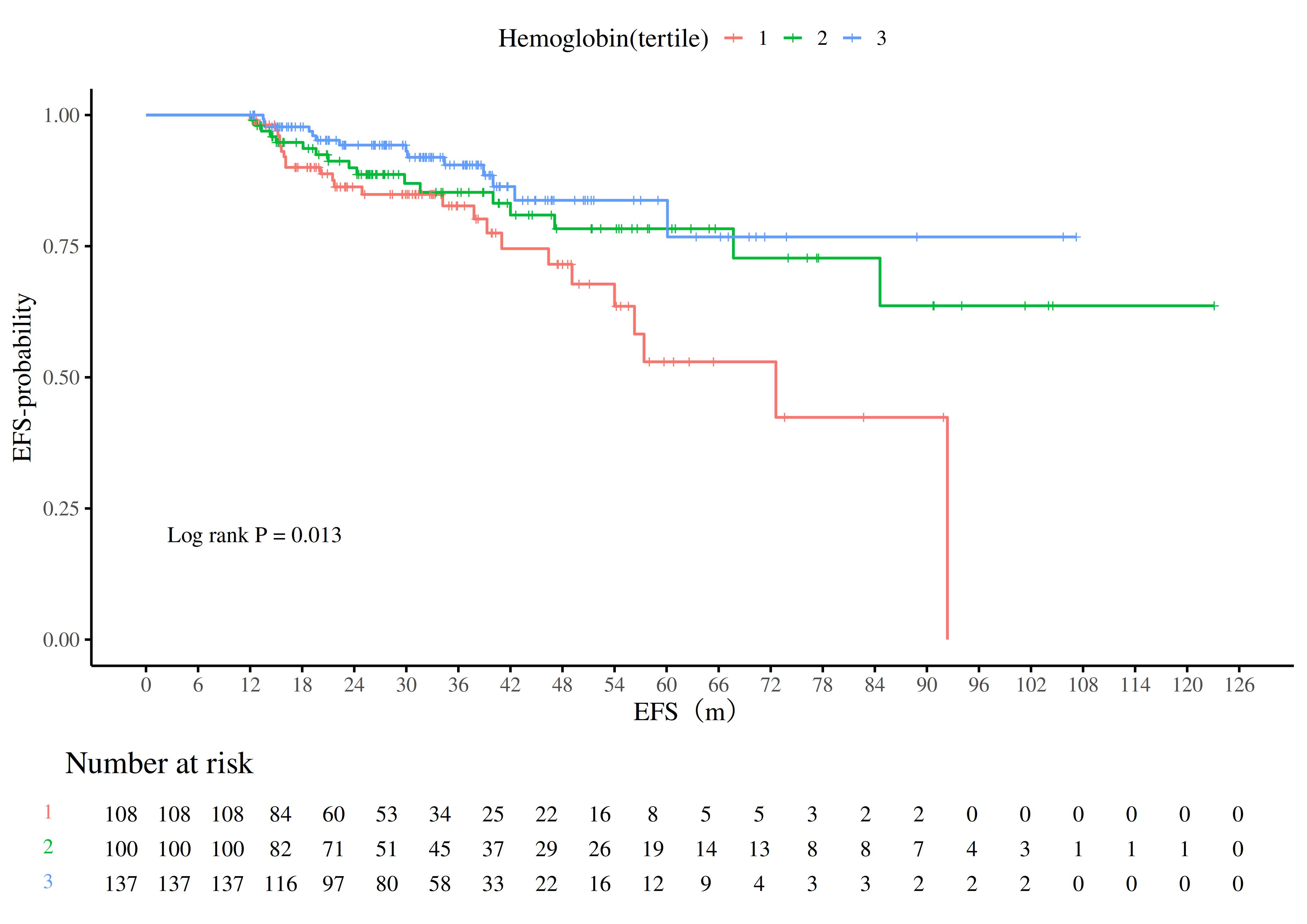

Supplement: Supplementary file 2 [file Image2.tiff]
