# Supplementary material for: Association between baseline hemoglobin levels and pathological complete response in 404 female breast cancer patients undergoing neoadjuvant therapy in Western Guangdong region: a retrospective cohort study
Source: Front Oncol. 2026 Mar 6;16:1699201. doi: 10.3389/fonc.2026.1699201 (PMC13002421; doi:10.3389/fonc.2026.1699201)
Supplement: Supplementary file 3 [file DataSheet1.pdf]

Supplementary table 1 Missing data characteristics and SMD

variable	level	type	n F	n M	Mean Y	Mean N	SD Y	SD N	Prop Y	Prop N	SMD
BMI	Normal	categorical	225	12					0.56	0.60	-0.09
BMI	Over	categorical	152	7					0.38	0.35	0.05
BMI	Under	categorical	27	1					0.07	0.05	0.07
			404	20							
CHD History	N	categorical	397	18					0.98	1.00	-0.14
CHD History	Y	categorical	7	0					0.02	0.00	0.14
			404	18							
Clinical Stage	I_IIA	categorical	33	0					0.08	0.00	0.30
Clinical Stage	IIB	categorical	94	3					0.23	0.16	0.18
Clinical Stage	IIIA	categorical	149	4					0.37	0.21	0.33
Clinical Stage	IIIB	categorical	41	5					0.10	0.26	-0.52
Clinical Stage	IIIC	categorical	87	7					0.22	0.37	-0.37
			404	19							
Diabetes History	N	categorical	389	20					0.96	0.95	0.06
Diabetes History	Y	categorical	15	1					0.04	0.05	-0.06
			404	21							
ER	0	categorical	165	9					0.41	0.45	-0.08
ER	1	categorical	239	11					0.59	0.55	0.08
			404	20							
Family Cancer History	N	categorical	399	21					0.99	1.00	-0.11
Family Cancer History	Y	categorical	5	0					0.01	0.00	0.11
			404	21							
HER2	0	categorical	86	4					0.21	0.20	0.03
HER2	1	categorical	52	7					0.13	0.35	-0.64
HER2	2	categorical	103	5					0.25	0.25	0.01
HER2	3	categorical	163	4					0.40	0.20	0.42
			404	20							
Hepatitis History	N	categorical	382	21					0.95	1.00	-0.25
Hepatitis History	Y	categorical	22	0					0.05	0.00	0.25
			404	21							
Hypertension History	N	categorical	371	19					0.92	0.90	0.05
Hypertension History	Y	categorical	33	2					0.08	0.10	-0.05
			404	21							
Menopausal Status	N	categorical	235	14					0.58	0.67	-0.17
Menopausal Status	Y	categorical	169	7					0.42	0.33	0.17
			404	21							
N	0	categorical	46	0					0.11	0.00	0.37
N	1	categorical	175	9					0.43	0.47	-0.08
N	2	categorical	96	2					0.24	0.11	0.31
N	3	categorical	87	8					0.22	0.42	-0.49
			404	19							
PR	0	categorical	203	7					0.50	0.35	0.30
PR	1	categorical	201	13					0.50	0.65	-0.30
			404	20							

Subtype	HR+/HER2+	categorical	81	0					0.20	0.00	0.51
Subtype	HR+/HER2-	categorical	164	12					0.41	0.63	
Subtype	HR-/HER2+	categorical	98	4					0.24	0.21	0.07
Subtype	HR-/HER2-	categorical	61	3					0.15	0.16	-0.02
			404	19							
T	1	categorical	21	0					0.05	0.00	0.24
T	2	categorical	193	4					0.48	0.21	0.54
T	3	categorical	134	10					0.33	0.53	-0.41
T	4	categorical	56	5					0.14	0.26	-0.35
			404	19							
Age		continuous	404	21	48.42	49.62	9.40	7.53			-0.13
Height		continuous	404	20	157.50	158.75	4.66	2.38			-0.27
KI67		continuous	404	16	44.94	37.50	21.72	21.06			0.34
Weight		continuous	404	21	57.55	59.55	8.65	8.79			-0.23

Abbreviations: BMI, body mass index; CHD, coronary heart disease; ER, estrogen receptor; HER2, human epidermal growth factor receptor 2; HR, hormone receptor; pCR, pathological complete response; PR, progesterone receptor; T, clinical tumour (TNM) stage; N, clinical nodal (TNM) stage; SMD, Standard Mean Difference; SD, Standard Deviation; Prop, proportion.

Notes: BMI, Under(<18.5 kg/m²), Normal (18.5–24.9 kg/m²), Over(⩾25.0 kg/m²). M represents the group of missing data, while F refers to the final included data group.

Supplementary table 2 The PH test results of the main variable and the adjustment variable

variable	Test statistic	p	-log2(p)
Age	4.31	0.04	4.72
BMI			
Normal	reference		
Over	0.04	0.84	0.25
Under	0.10	0.75	0.42
ER			
0	reference		
1	0.51	0.47	1.08
HER2			
0	reference		
1	0.15	0.70	0.51
2	0.12	0.73	0.45
3	0.04	0.83	0.26
**Hemoglobin**	**1.94**	**0.16**	**2.61**
Ki67	1.44	0.23	2.12
Menopausal Status			
N	reference		
Y	1.49	0.22	2.17
N			
0	reference		
1	0.27	0.61	0.72
2	1.07	0.30	1.73
3	0.30	0.58	0.78
PR			

	0	reference		
	1	0.09	0.76	0.39
T				
	1	reference		
	2	0.07	0.79	0.34
	3	0.00	0.97	0.05
	4	0.01	0.92	0.12

Notes: Hemoglobin was treated as a continuous variable. BMI, Under(<18.5 kg/m²), Normal (18.5–24.9 kg/m²), Over(⩾25.0 kg/m²).

Supplementary table 3 Detailed data of the subgroup analysis

Variables	n (%)	OR (95%CI)	P	P for interaction
All patients	404 (100.00)	1.02 (1.01 ~ 1.04)	0.005	
Menopausal Status				0.954
N	235 (58.17)	1.02 (1.00 ~ 1.04)	0.023	
Y	169 (41.83)	1.02 (1.00 ~ 1.05)	0.099	
BMI				0.834
Normal	225 (55.69)	1.02 (1.00 ~ 1.04)	0.061	
Over	152 (37.62)	1.02 (1.00 ~ 1.05)	0.048	
Under	27 (6.68)	1.04 (0.96 ~ 1.14)	0.328	
Stage				0.977
I_IIA	33 (8.17)	1.01 (0.96 ~ 1.07)	0.745	
IIB	94 (23.27)	1.02 (0.99 ~ 1.05)	0.177	
IIIA	149 (36.88)	1.02 (1.00 ~ 1.05)	0.077	
IIIB	41 (10.15)	1.01 (0.97 ~ 1.06)	0.536	
IIIC	87 (21.53)	1.01 (0.98 ~ 1.05)	0.410	
MS				0.939
HR-/HER2-	62 (15.35)	1.02 (0.98 ~ 1.06)	0.400	
HR-/HER2+	97 (24.01)	1.02 (1.00 ~ 1.05)	0.093	
HR+/HER2-	164 (40.59)	1.03 (0.99 ~ 1.07)	0.099	
HR+/HER2+	81 (20.05)	1.02 (0.99 ~ 1.05)	0.268	
Ki67				0.718
50<	220 (54.46)	1.02 (1.00 ~ 1.04)	0.084	
≥50	184 (45.54)	1.02 (1.00 ~ 1.05)	0.027	
Age				0.100
49<	195 (48.27)	1.01 (0.99 ~ 1.03)	0.338	
≥49	209 (51.73)	1.04 (1.01 ~ 1.06)	0.004	

Notes: Hemoglobin was treated as a continuous variable. BMI, Under(<18.5 kg/m²), Normal (18.5–24.9 kg/m²), Over(⩾25.0 kg/m²).

Supplementary table 4 Cox analysis of Hemoglobin and EFS

Factors	Crude model		Adjusted model	
	P	HR (95%CI)	*P*	HR (95%CI)
Hemoglobin	**<.001**	0.98 (0.96 ~ 0.99)	**<.001**	0.98 (0.96 ~ 0.99)
Hemoglobin Q3				
Q1(<123 g/L)		1.00 (Reference)		1.00 (Reference)
Q2(123~133 g/L)	**0.015**	0.54 (0.33 ~ 0.89)	**0.008**	0.49 (0.29 ~ 0.83)
Q3(>133g/L)	**<.001**	0.36 (0.20 ~ 0.65)	**<.001**	0.35 (0.19 ~ 0.64)
P for trend	**<.001**	0.96 (0.94 ~ 0.98)	**<.001**	0.96 (0.94 ~ 0.98)

Abbreviations: EFS, event-free survival; CI, confidence interval; HR, hazard ratio; Hb, hemoglobin.

Adjusted model: Adjusted for menopausal status, BMI, T stage, N stage, ER status, PR status, HER2 status, Ki67.

Notes: Hemoglobin tertile: Q1(<123 g/L), Q2(123~133 g/L), Q3(>133 g/L). BMI, Under(<18.5 kg/m²), Normal (18.5–24.9 kg/m²), Over(≥25.0 kg/m²).**Age was not adjusted in the Cox analysis.**

Supplementary table 5 Cox analysis after excluding the early censored cases

(A)

Factors	Crude model		Adjusted model	
	P	HR (95%CI)	*P*	HR (95%CI)
Hemoglobin	**0.006**	0.98 (0.96 ~ 0.99)	**0.005**	0.97 (0.96 ~ 0.99)
Hemoglobin Q3				
Q1(<123g/L)		1.00 (Reference)		1.00 (Reference)
Q2(123~133 g/L)	**0.032**	0.52 (0.28 ~ 0.95)	**0.006**	0.39 (0.20 ~ 0.76)
Q3(>133 g/L)	**0.012**	0.41 (0.21 ~ 0.83)	**0.014**	0.40 (0.19 ~ 0.83)
P for trend	**0.006**	0.97 (0.94 ~ 0.99)	**0.005**	0.96 (0.93 ~ 0.99)

(B)

Factors	Crude model		Adjusted model	
	P	HR (95%CI)	*P*	HR (95%CI)
Hemoglobin	**0.006**	0.98 (0.96 ~ 0.99)	**0.006**	0.97 (0.96 ~ 0.99)
Hemoglobin Q3				
Q1(<123g/L)		1.00 (Reference)		1.00 (Reference)
Q2(123~133 g/L)	**0.032**	0.52 (0.28 ~ 0.95)	**0.011**	0.42 (0.21 ~ 0.82)
Q3(>133 g/L)	**0.012**	0.41 (0.21 ~ 0.83)	**0.014**	0.41 (0.20 ~ 0.86)
P for trend	**0.006**	0.97 (0.94 ~ 0.99)	**0.005**	0.96 (0.94 ~ 0.99)

Abbreviations: CI, confidence interval; HR, hazard ratio; Hb, hemoglobin.

Adjusted model:(A) Adjusted for menopausal status, Age, BMI, T stage, N stage, ER status, PR status, HER2 status, Ki67.

(B) Adjusted for menopausal status, BMI, T stage, N stage, ER status, PR status, HER2 status, Ki67.

Notes: Hemoglobin tertile: Q1(<123 g/L), Q2(123~133 g/L), Q3(>133 g/L). BMI, Under(<18.5 kg/m²), Normal (18.5–24.9 kg/m²), Over(≥25.0 kg/m²).

Excluding the cases that were prematurely terminated from the study (a total of 59 cases were terminated within 12 months), 345 cases remained.

Supplementary Figure 1 Schoenfeld residual plot of hemoglobin

Notes: Hemoglobin was treated as a continuous variable.

Supplementary Figure 2 Kaplan Meier curves after excluding the early censored cases

Notes: Hemoglobin tertile: Q1(<123 g/L), Q2(123~133 g/L), Q3(>133 g/L). Excluding the cases that were prematurely terminated from the study (a total of 59 cases were terminated within 12 months), 345 cases remained.
